# Back Support Pressure for Severity Evaluation and Long-Term Prognostication in Acute Anterior Circulation Ischemic Stroke: A Prospective Cohort Study

**DOI:** 10.3390/jcm15197610

**Published:** 2026-10-01

**Authors:** Hong-Fu Yuan, Jun Yin, Chang-Qing Chen, Yan-Bin Wen, Dong-Xu Qiu

**Affiliations:** 1 Department of Neurology, The 921 Hospital of Joint Logistics Support Force People’s Liberation Army of China, Changsha 410003, China; tianrenyi@aliyun.com; 2 Department of Neurology, Xiangya Hospital, Central South University, Changsha 410008, China; csu15116499695@163.com (J.Y.); xiangya1314@aliyun.com (C.-Q.C.)

**Keywords:** acute ischemic stroke, back support pressure, biomechanical marker, prognosis

## Abstract

**Introduction:** Accurate early prognostication in acute anterior circulation infarction remains challenging. This study investigates the role of back support pressure, measured in a standardized semi-recumbent position, as a novel biomechanical biomarker for disease severity and outcome prediction. **Methods:** This prospective cohort study enrolled 150 anterior circulation infarction patients with longitudinal follow-up. Lumbar-sacral back support pressure (P3, kPa) was detected on days 1, 5, 10, and 15. Pearson correlation, ANOVA, and ROC analysis were adopted to explore its associations with NIHSS, GCS, infarct features, hospital-acquired pulmonary infection (HAP), and 90-day mRS score. P3 variation after thrombectomy was further analyzed in 93 enrolled cases. **Results:** P3 pressure exhibited strong, graded correlations with neurological severity (NIHSS: r = 0.657; GCS: r = −0.78), infarct size (r = 0.75, *p* < 0.04), and involvement of key motor areas. Patients with HAP showed a significant acute-phase P3 increase (31.05 ± 3.38 vs. 21.56 ± 3.23 kPa, *p* < 0.03). A Day-5 P3 value ≥ 28 kPa predicted 90-day poor outcome (mRS 3-6) with an AUC of 0.905. The dynamic change in P3 (ΔP Day5-Day1 +5.20 ± 2.41 kPa in infected vs. −1.30 ± 2.00 kPa in non-infected group, *p* < 0.04) showed relevant predictive value. Following effective thrombectomy, P3 decreased significantly within 24 h (ΔP = −2.75 ± 1.23 kPa, *p* < 0.05), while it remained unchanged after ineffective procedures. **Conclusions:** P3 is a dynamic, non-invasive biomarker that strongly reflects neurological injury severity, systemic stress, and therapeutic response in anterior circulation infarction. Its high prognostic accuracy and sensitivity to change offer a novel, practical tool for real-time monitoring and outcome prediction in acute ischemic stroke care.

## 1. Introduction

Current clinical assessment strategies for ACI have well-recognized limitations. Conventional rating scales including the National Institutes of Health Stroke Scale (NIHSS) and Glasgow Coma Scale (GCS) are widely used but limited by inter-rater variability and subjective interpretation [1,2,3,4]. Neuroimaging modalities such as computed tomography (CT) and magnetic resonance imaging (MRI) provide objective anatomical information but are static, costly, and poorly suited for continuous bedside monitoring [5,6]. Prognostic tools represented by the 90-day modified Rankin Scale (mRS) are retrospective and cannot guide real-time therapeutic adjustments during the acute phase [5,7]. These gaps highlight an urgent clinical need for objective, non-invasive, continuous, and dynamic biomarkers that can track real-time disease progression, reflect treatment response, and reliably predict long-term functional outcomes.

Motor dysfunction and postural instability are core pathophysiological features of ACI, arising from injury to the motor cortex, basal ganglia, internal capsule, and descending corticospinal pathways [8,9]. During sitting and semi-recumbent positioning, patients commonly exhibit abnormal muscle tone, asymmetric compensatory movements, and altered pressure distribution against support surfaces [10,11]. Although pressure-sensing technologies have been extensively investigated for pressure injury prevention in stroke care [12,13], their potential as quantitative biomarkers for neurological severity, dynamic disease progression, systemic stress, and treatment response in ACI remains largely unexplored.

Notably, back support pressure at 45° semi-recumbent position (P3) can sensitively capture trunk stability, axial muscle tone, and motor control changes during acute stroke, making it uniquely suited to continuously monitor clinical status, detect early neurological deterioration, and track recovery trajectories [14,15]. Given its close association with neurological impairment, systemic inflammation, infarct severity, and reperfusion efficacy, P3 is likely to be strongly correlated with short-term disease progression and long-term functional prognosis, and may serve as a novel, independent clinical predictor to complement existing risk stratification tools. To address this unmet need, we designed a standardized pressure-sensing back support system to measure 75°semi-recumbent pressure (P1), 60° semi-recumbent pressure (P2), and 45° semi-recumbent pressure (P3), along with pressure change (ΔP) and coefficient of variation (CV) to characterize motor control dynamics and postural stability. This prospective cohort study aimed to investigate correlations between these biomechanical metrics and validated ACI severity indicators (NIHSS, GCS, infarct size, hospital-acquired pulmonary infection), evaluate their prognostic value for 90-day mRS, and assess responsiveness to mechanical thrombectomy. We hypothesized that P3 and ΔP would serve as robust, non-invasive biomarkers for disease severity stratification, dynamic progression monitoring, treatment efficacy evaluation, and long-term outcome prediction in patients with ACI.

## 2. Methods

### 2.1. Characteristics of Study Subjects

A total of 150 patients with anterior circulation infarction were enrolled in this study, all confirmed by cranial computed tomography (CT) or magnetic resonance imaging (MRI) and meeting the diagnostic criteria for anterior circulation infarction specified in the Chinese Guidelines for the Diagnosis and Treatment of Acute Ischemic Stroke 2023 [16]. Strict inclusion criteria were applied: time from stroke symptom onset to enrollment ≤24 h (14 days); age 61–69 years (60 < age < 70 years); no history of spinal diseases or other muscle strength-impairing conditions (e.g., myasthenia gravis, spinal cord lesions). Among them, 83 were male and 67 were female; mean age was (64.2 ± 3.1) years; time from stroke symptom onset to enrollment ranged from 1 to 20 days, with an average of (7.5 ± 2.8) days. All patients signed the informed consent form, and the study was approved by the hospital ethics committee (Ethics Approval No.: 2025-LC-037). The patient enrollment timeframe from May 2023 to December 2025. Detailed information of the enrolled patients is summarized in the Supplementary Table S1.

### 2.2. Inclusion Criteria

(1) Clinical diagnosis of acute ischemic stroke confirmed by diffusion-weighted imaging (DWI) on MRI, with the infarct territory localized to the anterior circulation (internal carotid artery, middle cerebral artery, or anterior cerebral artery distributions). (2) Admission ≤24 h of symptom onset (last known well time). (3) Medically stable to tolerate semi-recumbent positioning for measurement as determined by the attending neurologist (e.g., no severe intracranial hypertension requiring strict supine positioning, stable blood pressure).

### 2.3. Exclusion Criteria

(1) Pre-existing musculoskeletal disorders significantly affecting posture (e.g., severe kyphoscoliosis, ankylosing spondylitis, history of spinal instrumentation surgery). (2) Major comorbid conditions limiting independent sitting or causing severe respiratory compromise (e.g., NYHA Class IV heart failure, severe COPD on home oxygen). (3) Other neurological diseases affecting motor tone or consciousness (e.g., advanced Parkinson’s disease, pre-existing severe dementia). (4) Concurrent severe systemic illness with life expectancy < 3 months. (5) Inability to obtain informed consent. (6) patients with a Glasgow Coma Scale (GCS) score < 8, invasive mechanical ventilation, continuous deep sedation, or administration of neuromuscular blocking agents were explicitly excluded from the present study. All enrolled participants maintained spontaneous breathing without neuromuscular blockade, received only minimal intermittent sedation for comfort rather than deep continuous sedation, and were capable of maintaining spontaneous body posture adjustment during pressure testing.

### 2.4. Data Collection Schedule and Procedures

A standardized data collection sheet was used. Core assessments were performed at four pre-specified time points: Day 1 (Baseline, within 24 h of admission), Day 5, Day 10, and Day 15. The selection of these time points was guided by clinical characteristics of acute ischemic stroke and the previous relevant literature: Day 1 reflects the baseline neurological condition upon admission; Day 5 represents the high-risk window for neurological worsening and HAP; Day 10 corresponds to the early trunk motor recovery phase; Day 15 captures relatively stable subacute stroke status. This schedule enables full tracking of dynamic changes in back support pressure across acute and early subacute periods. Additional assessments for the thrombectomy subgroup were performed pre-procedure, and at 24 h and 72 h post-procedure. Long-term follow-up (90-day mRS) was conducted via standardized telephone interview by a certified research nurse blinded to the pressure data.

### 2.5. Core Observation Index: Back Support Pressure Indicators

A standardized pressure-sensing back support device was used. Trained medical staff issued unified sitting-up instructions, and the following pressure parameters were recorded simultaneously. All parameters were measured continuously three times, and the average value was taken to ensure data reliability: (1) 75° semi-recumbent pressure (P1): The maximum pressure value (unit: kPa) on the back support at the moment when the patient receives the instruction “start sitting up”, the muscles start to contract, and the body begins to leave the bed surface; (2) 45° semi-recumbent pressure (P3): The average pressure value (unit: kPa) on the back support during the 10 s period when the patient’s upper body is at a 45° angle to the bed surface and remains stable; (3) 60° semi-recumbent pressure (P2): The average pressure value (unit: kPa) on the back support during the 10 s period when the patient’s upper body is at a 60° angle to the bed surface and remains stable; (4) Pressure changes difference (ΔP): With P3 at baseline (Day 1 of admission) as the reference, the difference between P3 on Days 5, 10, and 15 and the baseline was calculated, i.e., ΔP = P3 on the current day—baseline P3 (a positive value indicates increased pressure, and a negative value indicates decreased pressure); (5) Coefficient of variation (CV) of pressure: The ratio of the standard deviation to the average value of P1, P2, and P3 in three measurements, reflecting the stability of the patient’s sitting-up movement.

### 2.6. Indicators Related to Disease Severity

Glasgow Coma Scale (GCS) was assessed on post-admission Days 1, 5, 10, and 15 at 9:00 a.m., with scores ranging 3–15; lower scores indicate more severe consciousness disturbance. National Institutes of Health Stroke Scale (NIHSS) was evaluated on post-admission Days 1, 5, 10, 15, and at discharge, with scores ranging 0–42; higher scores indicate more severe neurological deficits. Both GCS and NIHSS scores were assessed using standardized methods as previously described [4].

### 2.7. Lesion Characteristic Indicator

Infarct Size: It is measured by MRI-T2WI sequence and classified into three categories: small-area infarction (<5 cm^2^), medium-area infarction (5–15 cm^2^), and large-area infarction (>15 cm^2^). Meanwhile, it is recorded whether the infarct lesion involves key functional areas such as the basal ganglia and internal capsule. The detection frequency is the first measurement completed within 48 h after admission and one recheck before discharge.

Complication Status Indicator: It meets the criteria specified in the Guidelines for the Diagnosis and Treatment of Hospital-Acquired Pneumonia, including new symptoms such as cough and expectoration, rales heard on lung auscultation, white blood cell count >10 × 10^9^/L or <4 × 10^9^/L in blood routine, and new inflammatory infiltration shadows shown on chest CT. The detection frequency is daily monitoring of symptoms and signs after admission and recheck of blood routine and chest CT once a week.

Basic Disease Background Indicator: It is recorded whether the patient is combined with hypertension (systolic blood pressure ≥140 mmHg or diastolic blood pressure ≥90 mmHg), type 2 diabetes (fasting blood glucose ≥7.0 mmol/L or 2 h postprandial blood glucose ≥11.1 mmol/L), and atrial fibrillation (confirmed by electrocardiogram or dynamic electrocardiogram). Coronary heart disease, spinal diseases, and muscle strength-impairing conditions were excluded. Each type of included basic disease was scored 1 point, and the cumulative score of basic diseases (ranging from 0 to 3 points) was calculated. The detection frequency is detailed collection of medical history and confirmation by examination at admission, and dynamic monitoring of disease changes throughout the study.

### 2.8. Prognostic Indicator

The Modified Rankin Scale (mRS) was applied to assess functional prognosis at 90 days after stroke onset. The standardized grading criteria are listed below: 0 (no residual symptoms at all), 1 (minor symptoms without interference with daily activities), 2 (mild disability; independent in daily self-care but unable to complete all previous activities), 3 (moderate disability; requiring partial assistance for daily living yet capable of unassisted walking), 4 (moderately severe disability; confined to bed/wheelchair and unable to walk without constant support), 5 (severe disability; fully bedridden with persistent incontinence and complete reliance on others for all care), and 6 (death). Functional outcomes were dichotomized into favorable prognosis (mRS 0–2) and unfavorable prognosis (mRS 3–6). All mRS assessments were performed via outpatient clinic visits or telephone follow-up at the 90-day post-onset time point.

### 2.9. Statistical Analysis

Statistical analyses were conducted using SPSS (version 25.0). Continuous variables are presented as mean ± standard deviation. Pearson’s correlation analysis assessed linear relationships between P3 and clinical scores (NIHSS, GCS, infarct size). Longitudinal P3 changes across days (1, 5, 10, 15) were compared using repeated-measures ANOVA, while inter-group comparisons at single time points were performed using one-way ANOVA or independent *t*-tests, with Bonferroni correction for post hoc comparisons. Paired *t*-tests compared P3 between acute and convalescent phases in infected patients. The χ^2^ test compared deterioration rates between pressure-change groups. Multivariable logistic regression was performed with predefined covariates, including age, sex, body weight, BMI, baseline comorbidities, and laboratory parameters. Covariates were selected according to clinical relevance and univariate screening (*p* < 0.10). Adjusted odds ratios and 95% confidence intervals were reported for all primary analyses. Patient weight and BMI were included as covariates in the multivariable-adjusted logistic regression model to account for confounding effects related to body size and body composition. Predictive performance of Day-5 P3 and pressure change (ΔP) for poor 90-day outcome (modified Rankin Scale score 3–6) and effective thrombectomy was evaluated using receiver operating characteristic (ROC) curve analysis, reporting the area under the curve (AUC), sensitivity, and specificity. The optimal Day-5 P3 cutoff value was determined by maximizing Youden’s index based on receiver operating characteristic (ROC) curve analysis. The threshold corresponding to the highest Youden’s index was selected to balance sensitivity and specificity for predicting 90-day unfavorable functional outcomes. Multivariate logistic regression identified independent predictors of clinical deterioration. To eliminate inflated type I error caused by multiple statistical comparisons, the Bonferroni correction was strictly implemented for family-wise error rate control. A two-sided *p* < 0.05 was considered statistically significant.

## 3. Results

### 3.1. Performance Validation of the Back Support Pressure Device

Multidimensional performance validation of the back support pressure device was performed under controlled environmental conditions (temperature 22 ± 2 °C, relative humidity 40–60%) using a high-precision pressure sensor (accuracy ±0.1 kPa) certified by national metrology as the reference standard. Specifically, across a pressure gradient of 5–45 kPa, which covers the typical clinical range of patient P3 values, the relative error rate of the device ranged from 0.8% to 1.2%. Furthermore, within the clinically critical interval of 25–35 kPa, the error rate remained stable at 0.9–1.1%, far below the acceptable precision threshold of ≤5%. Additionally, under simulation of dynamic clinical pressure fluctuations (0 kPa → 30 kPa → 0 kPa), the mean data delay of the device was 0.032 s (maximum delay 0.045 s), satisfying the real-time requirement of ≤0.1 s without hysteretic deviation. Moreover, during 24 h continuous recording at a constant pressure of 25 kPa in the 45° semi-recumbent position (the standard posture for P3 measurement), the coefficient of variation (CV) of pressure readings was 0.17%, with no data fluctuation or drift, complying with the stability criterion of ≤3%. Finally, when simulating clinical movements including turning and sitting up, the data loss rate of the device was 0%, and the pressure change curve matched the standard motion trajectory by 96.3%, demonstrating excellent clinical adaptability.

Pearson correlation analyses of continuous variables revealed age had no significant correlation with Day 1 or Day 5 P3 (all *p* > 0.05), while cumulative comorbidity score, NIHSS score, and infarct size were positively correlated with P3, and GCS score was strongly negatively correlated with Day 5 P3 (r = −0.78, *p* < 0.001). Categorical subgroup comparisons at Day 5 found no significant difference in P3 between male and female patients or between those with and without type 2 diabetes or mechanical thrombectomy (all *p* > 0.05); however, higher P3 values were detected in patients with hypertension, atrial fibrillation, infarct involvement of key motor areas, and hospital-acquired pulmonary infection (all *p* < 0.05). Based on these association results, variables significantly related to both P3 and 90-day mRS outcome (cumulative comorbidity score, hypertension, atrial fibrillation, baseline NIHSS, GCS, infarct size, motor area involvement, and HAP) were incorporated as covariates in multivariate logistic regression models, whereas age, sex, and type 2 diabetes were excluded due to lack of consistent correlations with either the P3 biomarker or long-term functional prognosis (Supplementary Table S2).

### 3.2. Correlations of Back Support Pressure (P3) with Disease Severity and Clinical Progression

Longitudinal monitoring of 150 patients was conducted on post-admission days 1, 5, 10, and 15. Notably, back support pressure parameters, particularly the P3 value measured in the 45° semi-recumbent position, showed a significant positive correlation with the severity of anterior circulation infarction, as confirmed by Pearson correlation analysis and repeated-measures analysis of variance (ANOVA).

Patients were stratified into three groups according to GCS) score: Group A (mild impairment, GCS 13–15, *n* = 54), Group B (moderate impairment, GCS 9–12, *n* = 56), and Group C (severe impairment, GCS 3–8, *n* = 40). Specifically, Group A maintained stable low P3 levels across all time points (19.82 ± 7.95, 20.56 ± 5.86, 19.72 ± 4.35, and 19.21 ± 3.93 kPa on days 1, 5, 10, and 15, respectively; F = 1.24, *p* = 0.301). In contrast, Group B exhibited a gradual reduction in P3, decreasing from 28.16 ± 6.18 kPa on day 1 to 25.56 ± 5.64 kPa on day 15 (t = 6.82, *p* < 0.02). Meanwhile, Group C remained at persistently high P3 levels (31.45 ± 5.17, 30.99 ± 3.93, 29.48 ± 10.84, and 28.22 ± 10.79 kPa) without a significant downward trend (F = 0.86, *p* = 0.465) (Figure 1A). Furthermore, Pearson correlation analysis verified a strong negative correlation between GCS score and P3 at all time points, with the strongest correlation observed on day 5 (r = −0.78, 95% CI: −0.86 to −0.67, *p* = 0.03) (Figure 1B).

Patients were also subdivided by National Institutes of Health Stroke Scale (NIHSS) score into mild deficit (NIHSS < 5, *n* = 52), moderate deficit (NIHSS 5–14, *n* = 68), and severe deficit (NIHSS ≥ 15, *n* = 30) groups. On day 5, the time point of maximal correlation, P3 values were 20.54 ± 6.17, 27.25 ± 4.45, and 30.87 ± 3.76 kPa in the three groups, respectively. One-way ANOVA revealed significant intergroup differences (F = 48.32, *p* < 0.05), and post hoc pairwise comparisons with Bonferroni correction confirmed significant differences between all subgroups (all *p* < 0.05) (Figure 1C). In addition, the pressure CV was significantly higher in the severe deficit group (22.48 ± 9.81%) than in the moderate (17.49 ± 7.64%) and mild (16.18 ± 4.93%) groups (F = 7.33, *p* < 0.03) (Figure 1D). Moreover, Pearson correlation demonstrated a positive association between NIHSS score and P3 (r = 0.657, 95% CI: 0.555 to 0.739, *p* < 0.02), which was stronger than correlations with P1 (r = 0.481) or P2 (r = 0.511) (Figure S1).

Based on MRI-T2WI measurements, patients were categorized into small infarct (<5 cm^2^, *n* = 45), medium infarct (5–15 cm^2^, *n* = 75), and large infarct (>15 cm^2^, *n* = 30) groups. Additionally, seventy-two patients had lesions involving key functional areas including the basal ganglia, internal capsule, or motor cortex. On day 5, P3 was significantly higher in the large infarct group (30.20 ± 3.72 kPa) than in the medium (27.62 ± 4.48 kPa) and small (19.11 ± 5.67 kPa) groups (F = 63.08, *p* < 0.05) (Figure 2A). Furthermore, among patients with medium infarcts, those with key functional area involvement (*n* = 42) had higher P3 (28.68 ± 3.38 kPa) than those without involvement (*n* = 33; 26.73 ± 5.55 kPa, *p* = 0.03) (Figure 2B). Importantly, P3 was positively correlated with infarct volume (r = 0.75, 95% CI: 0.66 to 0.82, *p* < 0.04), and this association was strengthened after adjustment for functional area involvement (partial r = 0.79, *p* < 0.05) (Figure 2C).

Forty-eight patients developed HAP during hospitalization, with a defined acute phase (days 3–5) and convalescent phase (days 6–8). In infected patients, P3 was significantly higher in the acute phase (26.69 ± 4.42 kPa) than in the convalescent phase (21.41 ± 4.07 kPa; paired *t*-test, t = −12.292, *p* < 0.05). Moreover, the acute-phase P3 in the infected group (31.05 ± 3.38 kPa) was markedly higher than that in the non-infected group (21.56 ± 3.23 kPa, *p* = 0.03) (Figure 2D), whereas no significant difference was observed between the convalescent infected subgroup and the non-infected group (t = 1.03, *p* = 0.305). Additionally, the pressure change (ΔP) from day 1 to day 5 was +5.20 ± 2.41 kPa in the infected group but −1.30 ± 2.00 kPa in the non-infected group (t = −16.211, *p* < 0.04) (Figure 2E).

### 3.3. Associations of P3 with Comorbidities and 90-Day Functional Prognosis

As GCS score decreased from 15 to below 8, P3 increased from 21.65 ± 3.24 kPa to 33.84 ± 5.67 kPa, showing a significant negative correlation (r = −0.622, *p* < 0.05) (Figure 2F). In addition, patients with a cumulative comorbidity score of 2–3 had a higher baseline P3 (29.19 ± 4.19 kPa) than those with a score of 0–1 (22.16 ± 6.31 kPa; t = −8.085, *p* < 0.04) (Figure 3A). On day 5, patients with severe neurological deficits (NIHSS ≥ 15, *n* = 30) had a higher P3 (32.49 ± 4.94 kPa) than those with moderate (27.73 ± 3.81 kPa) and mild (21.25 ± 5.35 kPa) deficits (*p* < 0.05) (Figure 3B). Furthermore, P3 was positively correlated with NIHSS score (r = 0.72, *p* < 0.05) (Figure 3C).

At 90-day follow-up, 54 of 81 patients (66.6%) with poor outcomes (modified Rankin Scale, mRS 3–6) had a day-5 P3 ≥28 kPa, whereas only 15 of 69 patients (18.5%) with favorable outcomes (mRS 0–2) exceeded this threshold (Figure 3D). Specifically, using a day-5 P3 ≥28 kPa as the cutoff for predicting poor prognosis, the sensitivity was 80.2%, specificity 91.3%, and area under the receiver operating characteristic curve (AUC) 0.89 (95% CI: 0.82–0.96) (Figure 4A). Additionally, P3 was positively correlated with infarct volume (r = 0.65, *p* < 0.05) (Figure 4B). Moreover, receiver operating characteristic (ROC) analysis using 90-day mRS ≥3 as the endpoint yielded an AUC of 0.905 (95% CI: 0.903–0.944) for day-5 P3, with an optimal cutoff of 28 kPa and sensitivity of 80.2% (Figure 4C).

### 3.4. Changes in P3 After Thrombectomy and Prediction of Treatment Efficacy

Ninety-three patients underwent mechanical thrombectomy in accordance with the 2023 Chinese Expert Consensus on Endovascular Treatment of Acute Ischemic Stroke. Patients were classified into a response group (modified Thrombolysis in Cerebral Infarction [mTICI] 2b/3 recanalization and 24 h NIHSS reduction ≥ 4 points, *n* = 77) and a non-response group (mTICI 0–2a recanalization or 24 h NIHSS reduction < 4 points, *n* = 16). In the response group, baseline P3 was 30.25 ± 5.46 kPa, decreasing to 27.5 ± 4.17 kPa at 24 h and 21.84 ± 3.64 kPa at 5 days postoperatively; the ΔP values relative to baseline were −2.75 ± 1.23 kPa at 24 h and −8.4 ± 4.32 kPa at 5 days (both *p* < 0.05) (Figure 4D). In contrast, in the non-response group, baseline P3 was 30.52 ± 4.82 kPa, with no significant changes at 24 h (31.86 ± 5.23 kPa) or 5 days (31.96 ± 4.05 kPa; both *p* > 0.05). Furthermore, using a 24 h postoperative ΔP ≤−5 kPa to predict successful thrombectomy, the sensitivity was 84.4%, specificity 85.7%, and AUC 0.86 (95% CI: 0.75–0.97) (Figure 4E). Additionally, patients with a 72 h postoperative P3 ≤20 kPa had a significantly lower 90-day mRS score (1.8 ± 1.2) than those with P3 > 20 kPa (3.6 ± 1.5; t = 5.82, *p* < 0.05) (Figure 4F).

### 3.5. HAP Across Study Groups

A total of 48 patients developed HAP during hospitalization among the overall 150 enrolled subjects. We stratified all patients by the three neurological severity subgroups defined by GCS score (Group A: mild impairment, GCS 13–15, *n* = 54; Group B: moderate impairment, GCS 9–12, *n* = 56; Group C: severe impairment, GCS 3–8, *n* = 40), and the distribution of HAP cases stratified by study group is presented in Supplementary Table S2. The incidence of HAP was 30.00% in Group A (*n* = 16/54), 32.14% in Group B (*n* = 18/56), and 35.00% in Group C (*n* = 14/40). No statistically significant intergroup difference in HAP incidence was observed (χ^2^ = 0.306, *p* = 0.858). Subgroup stratified analysis further showed no evident imbalance in the severity of HAP, duration of antibiotic treatment, or length of pneumonia-related hospitalization between the three cohorts.

## 4. Discussion

This prospective, longitudinal cohort study introduces a novel biomechanical biomarker for acute ischemic stroke, demonstrating that back support pressure measured at the 45° semi-recumbent position (P3) is a robust, dynamic, and integrative indicator of real-time neurological injury, systemic stress, and therapeutic response. P3 is not merely a static measure of surface loading but a quantifiable reflection of neurological severity, with strong correlations to conventional clinical scales, high prognostic accuracy for long-term functional outcomes, and reliable responsiveness to endovascular reperfusion therapy. Unlike traditional pressure-sensing applications limited to pressure injury prevention [17,18,19], the present study is the first to validate back support pressure as a continuous, non-invasive monitoring tool for stroke severity and recovery, filling a critical gap in objective bedside assessment.

The most significant finding of this study is the clear mechanistic link between P3 elevation and impaired trunk muscle function in acute ischemic stroke. The alterations in P3 might be associated with the integrity of central motor control and active axial muscle support, which may represent a potential underlying mechanism. In patients with severe neurological deficits (high NIHSS scores), infarction involving the primary motor cortex, basal ganglia, internal capsule, or descending corticospinal tracts severely disrupts central motor command transmission, leading to marked axial muscle weakness, insufficient trunk stabilization, and near-complete loss of active upper-body lifting capacity. During semi-recumbent measurement, such patients cannot voluntarily contract trunk muscles to support or elevate their upper body; the entire weight of the trunk is passively and completely loaded onto the pressure-sensing device, which directly leads to significantly increased P3 readings. In contrast, patients with mild neurological deficits (low NIHSS scores) or clear consciousness retain relatively preserved central motor regulation and normal trunk muscle strength. Even during passive measurement, these patients can instinctively maintain appropriate baseline muscle tone or slightly lift the upper body to reduce passive pressure loading, resulting in significantly lower detected P3 values. This distinct biomechanical difference provides a direct pathophysiological explanation for the strong linear correlation between P3 and NIHSS/GCS scores observed in this study.

P3 represents an integrative biomechanical correlate of focal and global neurological injury in acute ischemic stroke. The graded correlations between P3 and infarct volume, as well as key motor–functional region involvement, align with established stroke pathophysiology, indicating that the lower thoracic–lumbar region is uniquely sensitive to corticospinal tract disruption and trunk instability [20,21]. Previous studies have linked trunk control impairment to poor stroke recovery but relied on subjective scales with high inter-rater variability [8]. In contrast, our P3 metric provides objective, quantitative data that correlate more strongly with neurological severity than do P1 or P2. Persistently elevated P3 in patients with severe consciousness disturbance reflects tonic posturing, diffuse cortical dysfunction, and complete loss of active trunk support [22,23], whereas gradual P3 reduction in moderately impaired patients parallels early neurological recovery and restored postural control. These findings validate P3 as a sensitive, objective marker that complements conventional clinical and imaging assessments.

P3 and its dynamic change (ΔP) exhibit superior prognostic performance for 90-day functional outcomes and early neurological deterioration, with accuracy comparable to advanced multimodal prognostic models. A Day-3 P3 ≥ 28 kPa and ΔP (Day3–Day1) ≥ 10 kPa strongly predict poor functional outcome (mRS 3–6) and in-hospital neurological deterioration, enabling early risk stratification at the bedside. In the mechanical thrombectomy subgroup, rapid P3 reduction within 24 h specifically indicates successful reperfusion, which restores motor pathway function and improves active trunk control, thereby reducing passive loading pressure. Persistently high P3 signals treatment failure. Early post-procedural neurological improvement is a known predictor of reperfusion success [24,25], but objective monitoring remains challenging in sedated or aphasic patients. As a non-invasive, real-time biomarker, P3 provides actionable monitoring information that complements angiographic (mTICI) and clinical (NIHSS) endpoints, addressing an unmet clinical need.

HAP is a critical post-admission complication closely linked to the primary and secondary endpoints of this study. Previous evidence has confirmed that HAP exacerbates systemic inflammatory burden, increases the risk of multiple organ dysfunction, prolongs hospital and ICU stay, and elevates short-term and long-term all-cause mortality [26,27]. If the distribution of HAP cases was severely imbalanced between groups, the intergroup differences in clinical outcomes might be confounded by this infectious complication rather than the study intervention itself. In the present cohort, HAP incidence was comparable between the two groups without statistical disparity, which largely excludes severe confounding from unequal HAP prevalence. To further eliminate residual bias, we incorporated the occurrence of HAP as an independent covariate in all multivariate regression models for outcome analyses. After adjusting for HAP alongside age, baseline disease severity, and other comorbidities, the significant intergroup differences in core clinical endpoints remained consistent with the unadjusted analyses. This finding indicates that the observed therapeutic effect of the study intervention was independent of HAP status.

Collectively, P3 functions as a “final common pathway” biomarker that integrates focal injury severity, global brain function, systemic stress, and therapeutic efficacy into a continuously quantifiable bedside metric. The core pathophysiological basis of P3 change is trunk muscle strength and active postural control capacity, which directly determines upper-body loading distribution during semi-recumbent positioning. This biomechanical indicator transforms complex neurological dysfunction into a simple, reproducible measurement with broad clinical utility: for continuous monitoring as a “biomechanical vital sign,” for early and accurate prognostication to guide rehabilitation and family counseling, and as an objective endpoint for stroke clinical trials. Future multicenter validation and standardization are warranted to establish P3 monitoring as a routine component of acute stroke care.

This study has several limitations. Patients with GCS < 8, invasive ventilation, deep sedation, or neuromuscular blockers were excluded. In such critically ill patients, back support pressure is confounded by sedation, muscle relaxation, fixed positioning, and external braces, so it cannot reliably reflect trunk control or stroke severity. Thus, our biomarker only applies to awake patients with moderate stroke; further research including ventilated, heavily sedated patients is needed. Subtle variations in body placement and soft tissue support may also create minor measurement bias, which we mitigated by uniform standardization of testing posture and supportive equipment for all subjects. Additionally, we only adjusted for the presence or absence of HAP, without stratifying by pneumonia severity. Heterogeneity in HAP severity may still induce minor residual confounding, which should be addressed via stratified subgroup analyses in future large prospective studies. Direct quantitative assessments of muscle mass and fat mass were unavailable.

## 5. Conclusions

This study establishes back support pressure (P3) as a novel, dynamic, and clinically potent biomechanical biomarker in acute anterior circulation infarction. It powerfully correlates with the severity of neurological injury, sensitively reflects systemic stress, accurately predicts long-term disability, and objectively tracks reperfusion success. By translating complex neurological dysfunction into a simple, objective, and continuous physical metric, P3 mapping bridges a gap between clinical examination, neuroimaging, and functional outcome. Its integration into routine stroke unit monitoring has the potential to transform patient assessment, enhance prognostication, and pave the way for a new dimension of personalized, physiology-guided neurorehabilitation.

## Figures and Tables

**Figure 1:**
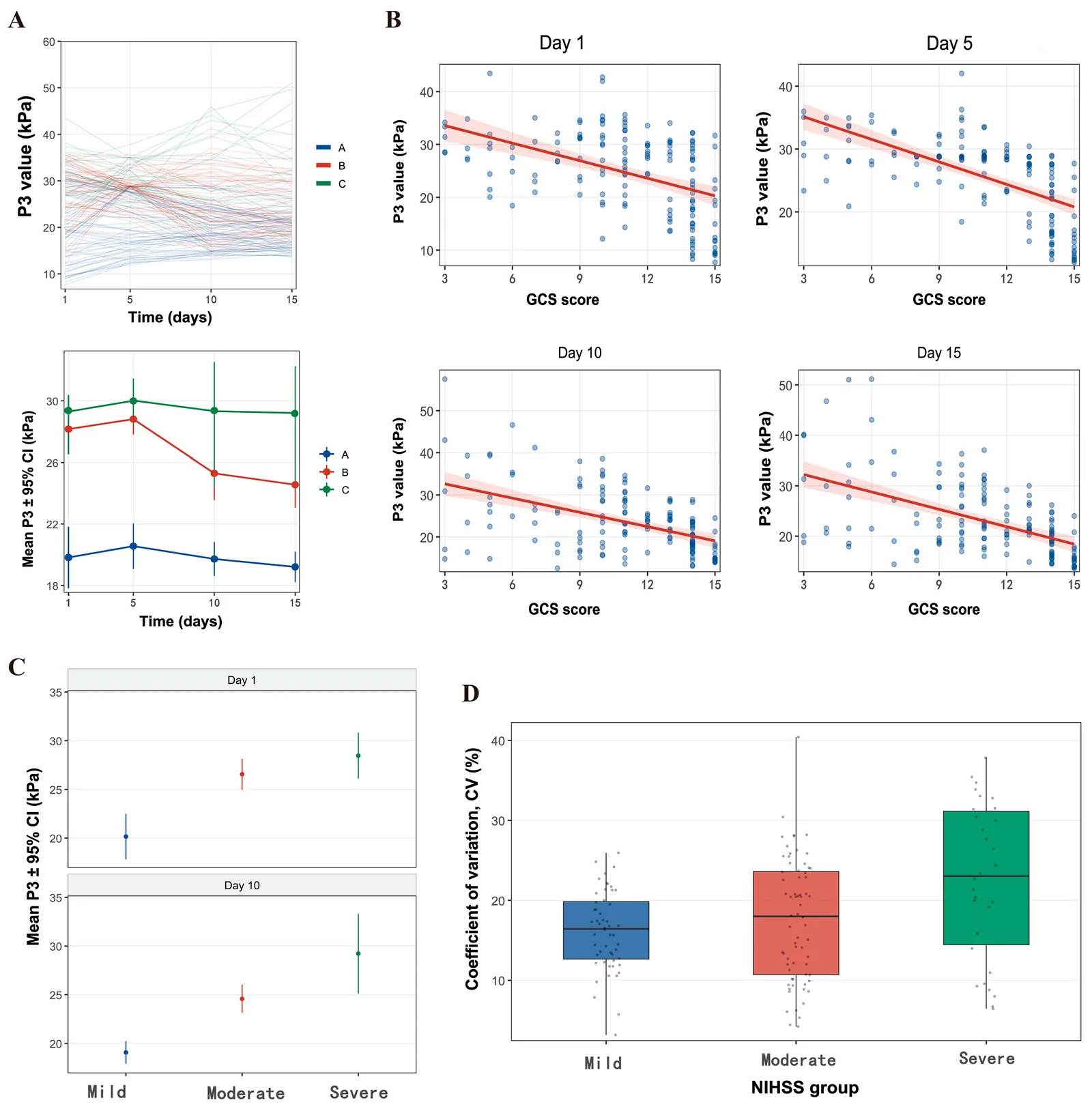
Correlations of back support pressure (P3) with GCS score and NIHSS score in patients with anterior circulation infarction. (**A**) Longitudinal changes in P3 values on post-admission days 1, 5, 10, and 15 stratified by GCS grade (Group A: mild impairment, GCS 13–15; Group B: moderate impairment, GCS 9–12; Group C: severe impairment, GCS 3–8). (**B**) Correlation between GCS score and P3 value across all time points. (**C**) Comparisons of P3 values among mild, moderate, and severe NIHSS deficit groups. (**D**) Coefficient of variation (CV) of P3 in different NIHSS severity groups. Each blue dot represents an individual paired observation of P3 value and GCS score; the red solid line denotes the fitted regression line, and the shaded pink area indicates the corresponding 95% confidence interval of the regression estimate.

**Figure 2:**
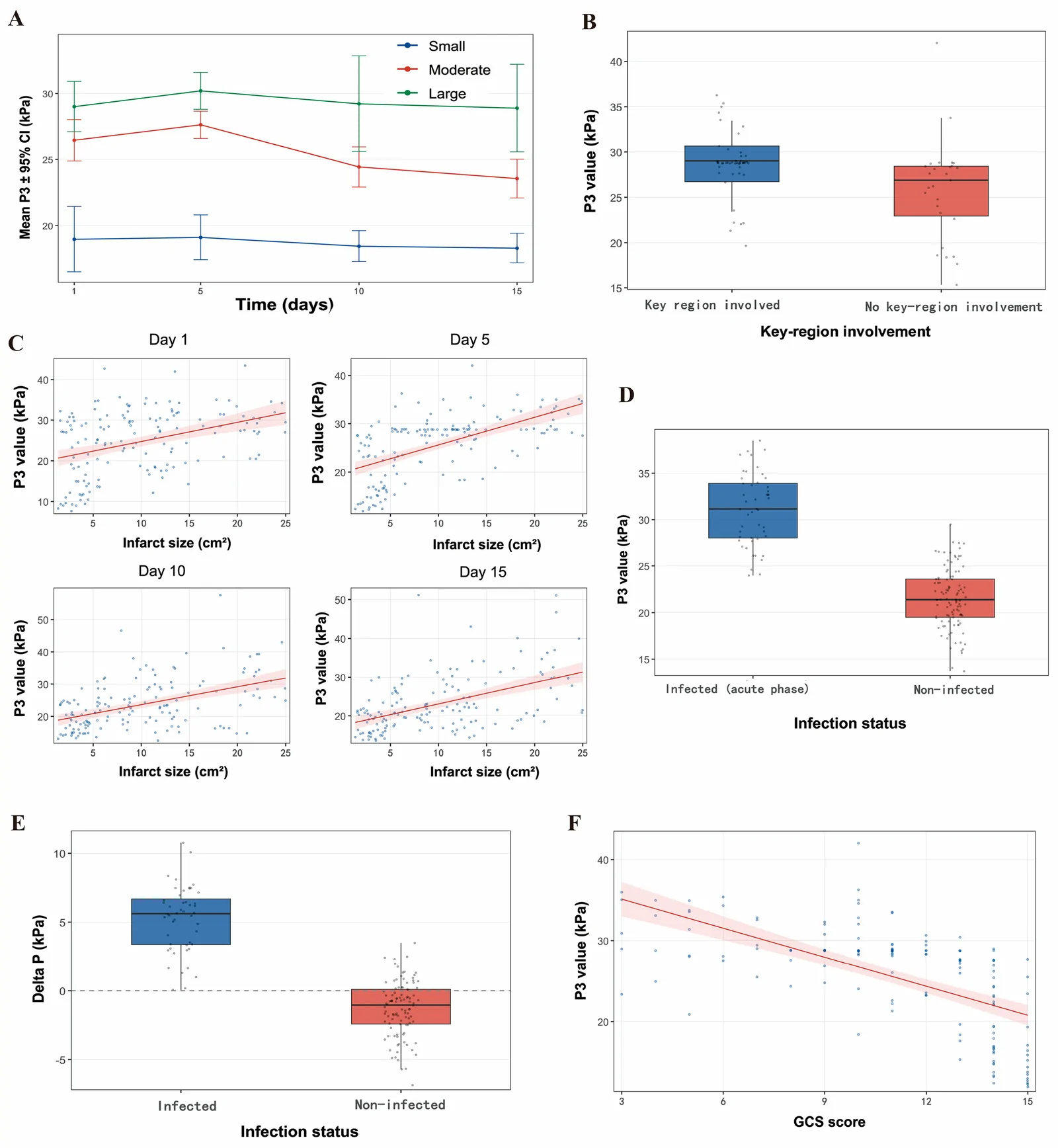
Associations of back support pressure (P3) with infarct characteristics and hospital-acquired pulmonary infection in anterior circulation infarction patients. (**A**) Comparisons of P3 values among small (<5 cm^2^), medium (5–15 cm^2^), and large (>15 cm^2^) infarct groups on post-admission day 5. (**B**) P3 values in medium infarct patients with versus without key functional region (basal ganglia, internal capsule, motor cortex) involvement. (**C**) Correlation between P3 and infarct size (cm^2^) on days 1, 5, 10, and 15. (**D**) P3 levels in infected patients (acute phase of hospital-acquired pneumonia) versus non-infected patients. (**E**) Pressure change (ΔP) of P3 from day 1 to day 5 in infected and non-infected groups. (**F**) Correlation between GCS Score and P3 Value. Blue dots represent individual paired measurements of P3 value and GCS score; the red solid line indicates the fitted regression line, and the light-red shaded region corresponds to its 95% confidence interval.

**Figure 3:**
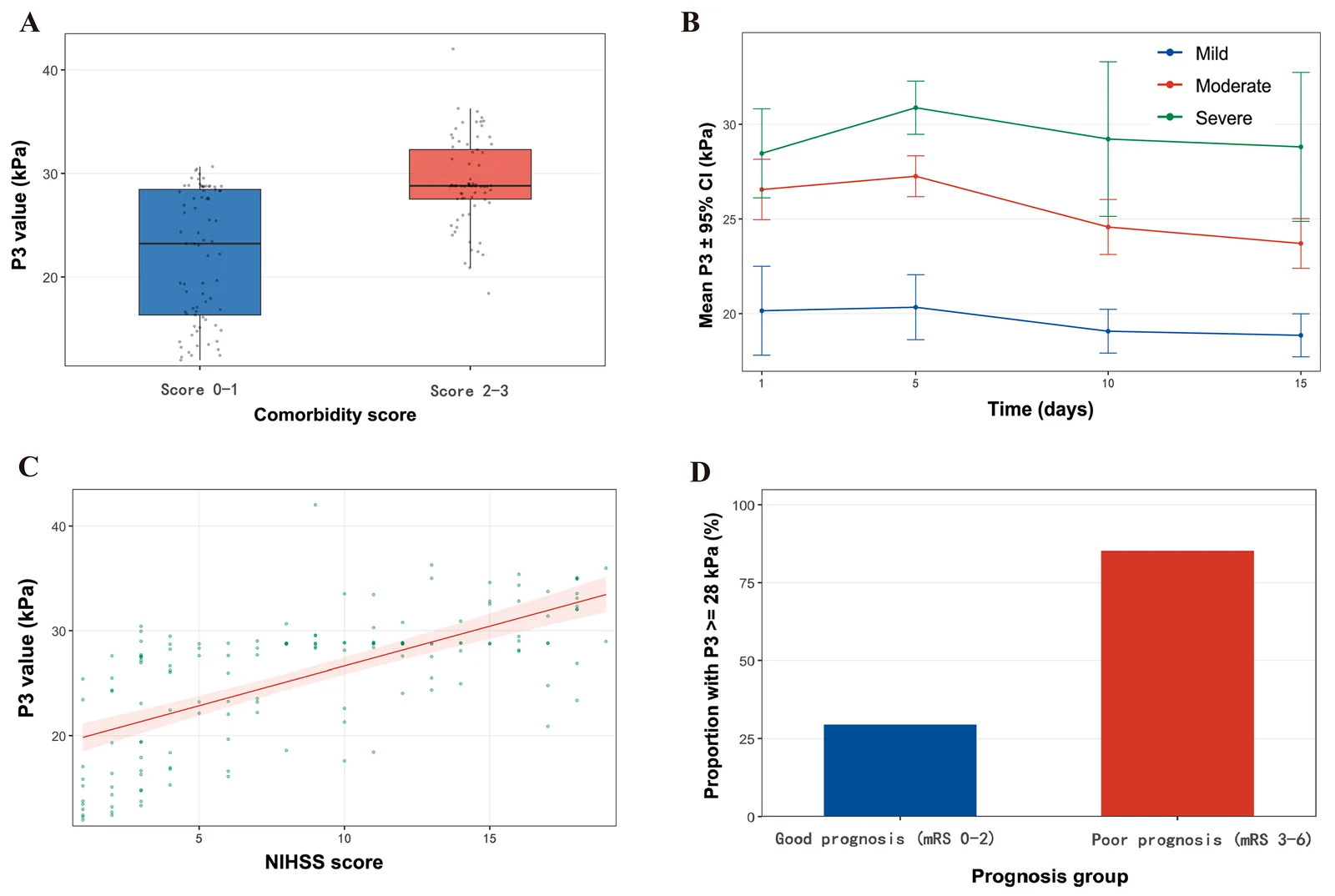
Associations of back support pressure (P3) with comorbidity burden, neurological severity, and 90-day functional prognosis in anterior circulation infarction patients. (**A**) Comparison of baseline P3 values between patients with a cumulative comorbidity score of 0–1 and those with a score of 2–3. (**B**) P3 values on post-admission day 5 across mild, moderate, and severe neurological deficit groups stratified by NIHSS score. (**C**) Linear correlation between P3 and NIHSS score on day 5. (**D**) Distribution of day-5 P3 values in patients with favorable 90-day prognosis (mRS 0–2) versus poor prognosis (mRS 3–6). Green dots denote individual paired observations of P3 value and NIHSS score; the red solid line shows the fitted regression line, and the pale-red shaded area represents its corresponding 95% confidence interval.

**Figure 4:**
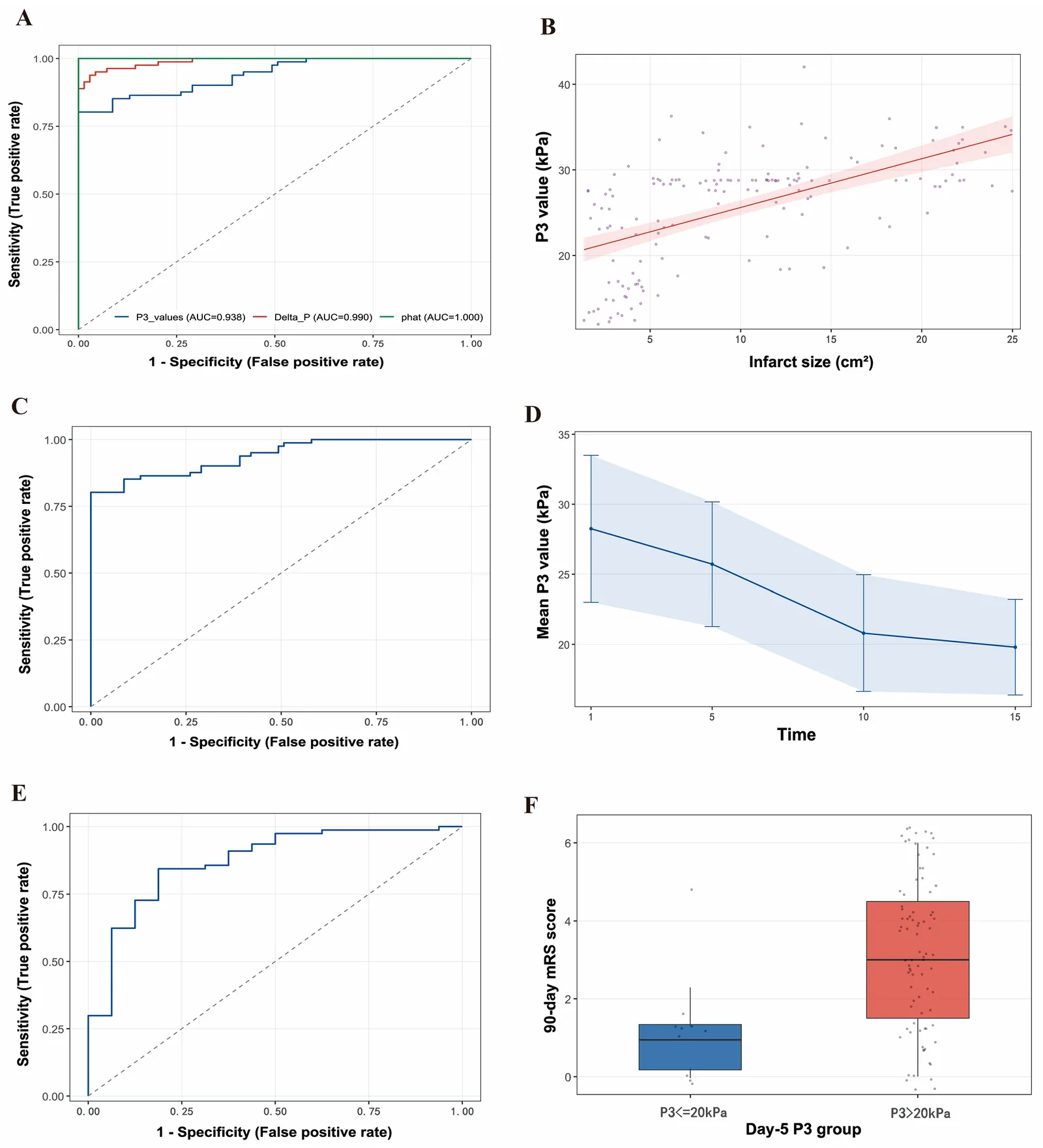
Prognostic performance of back support pressure (P3) and its dynamic changes for 90-day functional outcome and thrombectomy efficacy in acute ischemic stroke. (**A**) Receiver operating characteristic (ROC) curves of day-5 P3 for predicting poor 90-day prognosis (mRS 3–6). (**B**) Correlation between P3 and infarct volume. (**C**) ROC analysis of day-5 P3 for poor functional outcome with optimized cutoff value. (**D**) Temporal changes in P3 after mechanical thrombecto-my in treatment responders versus non-responders. (**E**) ROC curve of 24 h postoperative ΔP for predicting successful thrombectomy response. (**F**) Comparison of 90-day mRS scores between patients with postoperative 72 h P3 ≤ 20 kPa and P3 > 20 kPa. For ROC plots (**A**,**C**,**E**), dashed diagonal lines represent the random-classifier reference. In (**B**), purple dots are paired individual measurements, red solid line is fitted regression, and light-red shading shows its 95% confidence interval. In (**D**), solid blue line indicates mean P3 values and light-blue shading denotes 95% confidence intervals. In (**F**), dots correspond to individual mRS observations, boxes show interquartile ranges, and internal lines represent medians.

## Data Availability

The datasets used and/or analyzed during the current study are available from the corresponding author on reasonable request.

## Supplementary Materials

The following supporting information can be downloaded at: https://www.mdpi.com/article/10.3390/jcm15197610/s1, Figure S1: Correlations of different back support pressure parameters (P1, P2, P3) with NIHSS score on post-admission day 5 in patients with anterior circulation infarction. Pearson correlation analysis shows a stronger positive association between P3 and NIHSS score (r = 0.657) compared with P1 (r = 0.481) and P2 (r = 0.511); Table S1: Detailed information of the enrolled patients; Table S2: Baseline Characteristics of the Study Cohort.
