# Supplementary material for: Back Support Pressure for Severity Evaluation and Long-Term Prognostication in Acute Anterior Circulation Ischemic Stroke: A Prospective Cohort Study
Source: J Clin Med. 2026 Oct 1;15(19):7610. doi: 10.3390/jcm15197610 (PMC13666452; doi:10.3390/jcm15197610)
Supplement: Supplementary file 1 [file jcm-15-07610-s001.zip › Figure S1.pdf]

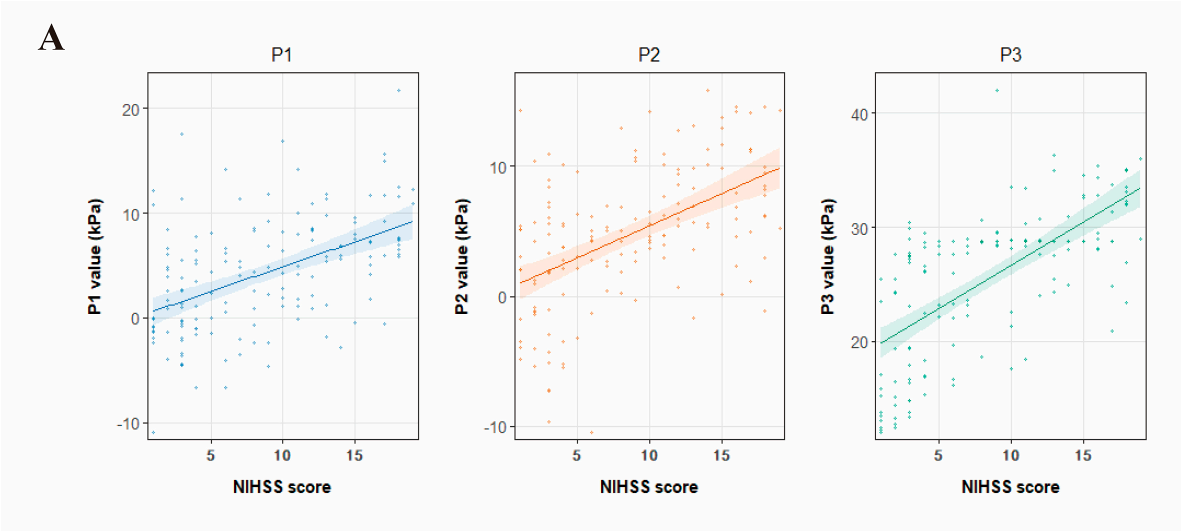

Figure S1. Correlations of different back support pressure parameters (P1, P2, P3) with NIHSS score on post-admission day 5 in patients with anterior circulation infarction. Pearson correlation analysis shows a stronger positive association between P3 and NIHSS score (r=0.657) compared with P1 (r=0.481) and P2 (r=0.511).
