# Supplementary material for: Back Support Pressure for Severity Evaluation and Long-Term Prognostication in Acute Anterior Circulation Ischemic Stroke: A Prospective Cohort Study
Source: J Clin Med. 2026 Oct 1;15(19):7610. doi: 10.3390/jcm15197610 (PMC13666452; doi:10.3390/jcm15197610)
Supplement: Supplementary file 1 [file jcm-15-07610-s001.zip › Supplymentary Table S2.pdf]

Table S2. Baseline Characteristics of the Study Cohort

Characteristic	Overall (n=150)	Favorable (mRS 0–2, n=82)	Unfavorable (mRS 3–6, n=68)	P-value
**Anamnestic & Demographic Characteristics**				
Age, mean ± SD, years	64.8 ± 2.7	64.5 ± 2.6	65.1 ± 2.8	0.203
Male sex, n (%)	89 (59.3)	48 (58.5)	41 (60.3)	0.812
Onset-to-enrollment time, mean ± SD, hours	12.4 ± 3.1	12.1 ± 2.9	12.8 ± 3.3	0.187
Cumulative comorbidity score, mean ± SD (range 0–3)	1.2 ± 0.8	1.0 ± 0.7	1.5 ± 0.9	**<0.001**
Hypertension history, n (%)	112 (74.7)	56 (68.3)	56 (82.4)	**0.042**
Type 2 diabetes history, n (%)	45 (30.0)	21 (25.6)	24 (35.3)	0.215
Atrial fibrillation history, n (%)	38 (25.3)	16 (19.5)	22 (32.4)	0.061
**Clinical Neurological & Imaging Characteristics**				
Admission GCS score, mean ± SD	13.8 ± 1.2	14.5 ± 0.8	12.9 ± 1.3	**<0.001**
Baseline NIHSS score, mean ± SD	8.2 ± 3.5	5.1 ± 2.2	11.9 ± 3.1	**<0.001**
Infarct size classification, n (%)				**<0.001**
Small (<10 mL)	52 (34.7)	41 (50.0)	11 (16.2)	
Medium (10–30 mL)	65 (43.3)	35 (42.7)	30 (44.1)	
Large (>30 mL)	33 (22.0)	6 (7.3)	27 (39.7)	
Involvement of key motor areas, n (%)	71 (47.3)	29 (35.4)	42 (61.8)	**0.002**
Hospital-acquired pulmonary infection (HAP), n (%)	48 (32.0)	12 (14.6)	36 (52.9)	**<0.001**
**Biomedical Biomechanical Parameters**				
Day 1 P3 value, mean ± SD, mmHg	28.6 ± 4.2	26.9 ± 3.8	30.7 ± 4.5	**<0.001**
Day 5 P3 value, mean ± SD, mmHg	32.1 ± 5.3	28.8 ± 4.1	36.0 ± 5.0	**<0.001**
Day 1 ΔP (P3-P1), mean ± SD, mmHg	10.2 ± 3.1	9.1 ± 2.8	11.5 ± 3.2	**<0.001**
Day 5 ΔP (P3-P1), mean ± SD, mmHg	13.5 ± 4.0	11.2 ± 3.3	16.3 ± 3.8	**<0.001**
**Treatment-Related Characteristics**				
Mechanical thrombectomy received, n (%)	93 (62.0)	51 (62.2)	42 (61.8)	0.965
Thrombectomy mTICI grade, n (%)				**0.003**
0–2a (poor recanalization)	28 (30.1)	10 (19.6)	18 (42.9)	
2b–3 (good recanalization)	65 (69.9)	41 (80.4)	24 (57.1)	

Data are presented as mean ± SD or n (%). P-values in red indicate statistical significance (P < 0.05).

Abbreviations: mRS = modified Rankin Scale; GCS = Glasgow Coma Scale; NIHSS = National Institutes of Health Stroke Scale; HAP = hospital-acquired pneumonia; ΔP = pressure differential (P3-P1); mTICI = modified Thrombolysis in Cerebral Infarction.
